# Obesity, insulin resistance and their interaction on liver enzymes

**DOI:** 10.1371/journal.pone.0249299

**Published:** 2021-04-21

**Authors:** Chenbing Liu, Min Shao, Ling Lu, Chenzhao Zhao, Lihong Qiu, Zhong Liu

**Affiliations:** 1 Department of Health Management, The First Affiliated Hospital, Zhejiang University School of Medicine, Hangzhou, China; 2 Zhejiang Nutriease Health Technology Company Limited, Hangzhou, China; East Tennessee State University, UNITED STATES

## Abstract

**Introduction:**

To investigate weight status, insulin resistance assessed by HOMA-IR, and their interaction on liver function in non-diabetic Chinese adults.

**Methods and results:**

A total of 7066 subjects were included, and divided into normal weight (n = 3447), overweight (n = 2801), and obese (n = 818) groups. Data including weight, height, waist circumference, fasting blood glucose, fasting insulin, total cholesterol, triglycerides, y-glutamyl transferase (GGT), aspartate aminotransferase (AST), and alanine aminotransferase (ALT) were acquired. In multi-linear regression analysis for liver enzymes as dependent variables, insulin resistance emerged as a determinant of ALT (β = 0.165, *P*<0.001), AST (β = 0.040, *P*<0.001) and GGT (β = 0.170, *P*<0.001) after adjusting for age, sex, body mass index, triglyceride, and cholesterol. Interactions between insulin resistance and weight status by body mass index were observed in ALT (*P*<0.001), AST (*P*<0.001) and GGT (*P* = 0.0418).

**Conclusion:**

Insulin resistance had significant associations with greater risk of elevated ALT, AST and GGT level in non-diabetic Chinese adults, especially among those who were overweight/ obese.

## Introduction

The dramatic increase in obesity worldwide remains challenging. It has been estimated that by 2030 about 40% of world population will be overweight and 20% will be obese [1]. Obesity is a well-known risk factor for metabolic syndrome [1, 2], which can further lead to diabetes, cardiovascular diseases, and non-alcoholic fatty liver disease (NAFLD). A Lancet article on trends in adult body-mass index in 200 countries from 1975 to 2014 reported that in 2014, more obese men and women lived in China than in the United States, even for severe obesity [3]. Obesity remains a clinical and public health issue worldwide.

Insulin resistance is defined as a reduced sensitivity to the action of insulin, and a lower glucose uptake and utilization in the liver, being exacerbated by obesity and the intake of dietary fats [4, 5].Homeostasis Model Assessment of insulin resistance(HOMA-IR) is a common efficient way to assess insulin resistance [6], which has been proven to be equally good as the gold standard for measuring insulin resistance.

Overweight or obese individuals have higher risk of developing steatosis, causing abnormal liver function [7],while liver enzymes including alanine aminotransferase (ALT), aspartate aminotransferase (AST), and y-glutamyl transferase (GGT) are valid indicators of liver injury [8, 9]. Previous studies found associations between NAFLD and the presence of insulin resistance as well as metabolic syndrome including hypertension, type 2 diabetes and obesity [5, 10, 11]. The role of liver enzymes in predicting the development of type 2 diabetes was previously examined but the results were not consistent. Several studies addressed the prospective relation of ALT with type 2 diabetes [10, 12, 13]; some indicated GGT but not ALT remains a significant predictor of diabetes [14, 15]. Similarly, the association of insulin resistance and abnormal liver enzymes remained inconsistent. Some studies indicated that HOMA-IR increased as the levels of liver enzyme elevated; ALT and AST, not GGT showed a significant association with HOMA-IR [5, 16]. Overweight/obese individuals have increased fat accumulation that may be the primary leading cause to insulin resistance, which further contribute to steatosis, and even steaohepatitis by stimulating lipolysis and de novo lipogenesis. We hypothesized that the associations of insulin resistance with liver enzymes are modified by the obese status. Therefore, the aim of our study was to evaluate the associations of insulin resistance with liver enzymes and the modifying effect of weight status on these associations in non-diabetic Chinese adults. To test this hypothesis, we analyzed weight status, insulin resistance, and their interaction on liver function, as these findings could provide further evidence for improving clinical treatment.

## Materials and methods

This study recruited subjects from the southern part of China who underwent annual physical check-up in the Health Management Center, the First Affiliated Hospital, Zhejiang University School of Medicine from January 2017 to May 2019. Subjects’ data were collected on the same date when they underwent the physical check-up. The inclusion criteria were subjects aged 18–65 years, BMI ≥18.5. The exclusion criteria were subjects diagnosed with diabetes mellitus, or currently taking oral insulin or insulin injection. We also removed the duplicated data from the same subjects and kept the first data acquired in 2017. We accessed the record on Sept 2019 to obtain the retrospective data with a total of 7066 subjects were included. The study protocol was approved by the Clinical Research Ethics Committee of the First Affiliated Hospital, Zhejiang University School of Medicine. Authors have no access to information that coud identify individuals after data collection.

Body mass index was calculated as weight(kg)/height(m^2^) and categorized according to Asian population criteria [17]: 18.5 to 23.9 kg/m^2^(normal weight), 24.0 to 27.9 kg/m^2^(overweight), and greater or equal to 28 kg/m^2^(obese). Fasting serum glucose, glycated hemoglobin, triglyceride, total cholesterol, ALT, AST, and GGT were measured using standard laboratory methods. Fasting insulin was detect by chemiluminecent microparticle immunoassay(CMIA) [18]. Insulin resistance index (HOMA-IR) was calculated using the following formula: (fasting insulin U/ml x fasting glucose mmol/l)/22.5.

### Statistical analysis

Statistical analysis was performed using SPSS 20.0. Data are shown as means ± standard deviation values, otherwise indicated using interquartile range M (P_25,_P_75_) for non-normal distribution data. Comparisons of the means of variables between the three weight status groups (normal, overweight, obese) were determined by ANOVA. Chi square test was used for categorical analysis. Spearman correlation analysis was done to see the correlation of different variables after adjusting for age and sex. Multi-linear regression models were used to estimate the significance of liver enzymes including ALT, AST, GGT and HOMA-IR while adjusting for confounding factors. Interactions were performed by the “interactions” package R 3.6.3 to determine the interactions between different variables. A p-value <0.05 was considered significant.

## Results

In total, 7066 subjects aged 18–65 years were entered into the analysis. Table 1 presented the baseline characteristics of the subjects, divided into three groups on the basis of BMI: normal weight (n = 3447), overweight (n = 2801) and obese (n = 818). The prevalence of overweight and obese in males was higher than those in females. The median value of ALT, AST, and GGT was the highest in obese subjects when comparing to normal and overweight subjects. Table 2 showed that serum activity of three liver enzymes was positively correlated with BMI, waist circumference, fasting insulin and HOMA-IR after adjustment for age and sex. Serum ALT was correlated with BMI, waist circumference, fasting insulin and HOMA-IR more than AST and GGT. BMI presented the highest correlation with HOMA-IR (r = 0.499, *P*<0.001).

**Table 1:** Characteristics of subjects by weight status.

	Normal weight(n = 3447)	Overweight(n = 2801)	Obese(n = 818)	*p* value
Age (years)	46.68±9.723	48.38±8.676*	47.3±9.220*^#^	<0.001
BMI	21.828±1.436	25.736±1.112	29.939±2.002	
Sex				<0.001
Male	1536 (44.6)	2018 (72.0)	624 (76.3)	
Female	1911 (55.4)	783 (28.0)	194 (23.7)	
Waist (cm)	79.45±6.058	89.47±5.313*	98.77±6.857*^#^	<0.001
ALT (U/L)	16.00 (12,22)	22 (16,32)*	29(19,43) *^#^	<0.001
AST (U/L)	20.39±8.871	23.02±11.165*	25.69±13.715*^#^	<0.001
GGT (U/L)	18 (12,28)	29(19,49)*	38(24,60) *^#^	<0.001
HOMA-IR	1.303±0.715	1.982±1.28*	3.0436±1.88*^#^	<0.001
Fasting Glucose (mmol/L)	4.886±0.934	5.6713±0.765*	5.864±0.894*^#^	<0.001
Fasting Insulin(mIU/L)	5.916±2.918	8.536±4.285*	12.549±6.66*^#^	<0.001
Triglyceride (mmol/L)	1.378±0.985	2.152±1.470*	2.3995±2.069*^#^	<0.001
Cholesterol (mmol/L)	4.622±0.853	4.777±0.889*	4.803±0.910*	<0.001
HDL-cholesterol (mmol/L)	1.323±0.347	1.12±0.284*	1.044±0.251*^#^	<0.001
LDL-cholesterol (mmol/L)	2.636±0.706	2.795±0.747*	2.785±0.756*	<0.001

Data are means±SD, n (%), or median (interquartile range); body mass index (weight/height^2^) is calculated to determine the weight status; ALT: alanine aminotransferase; AST: aspartate aminotransferase; GGT: γ-glutamyltransferase

* compared with normal weight group was statistically significant

^#^ overweight compared with obese group was statistically significant. The Fisher’s Least Significant Difference (LSD) post hoc test was used to evaluate pairwise comparisons.

**Table 2:** Correlation of serum ALT, AST, GGT, HOMA-IR and Fasting Insulin with other variables adjusted for age and sex.

	ALT		AST		GGT		HOMA-IR		Fasting insulin	
	r	*p* value	r	*p* value	r	*p* value	r	*p* value	r	*p* value
BMI	0.256	<0.001	0.161	<0.001	0.177	<0.001	0.499	<0.001	0.515	<0.001
Waist	0.258	<0.001	0.163	<0.001	0.196	<0.001	0.481	<0.001	0.489	<0.001
Glucose	0.104	<0.001	0.061	<0.001	0.120	<0.001	0.416	<0.001	0.154	<0.001
HOMA-IR	0.268	<0.001	0.160	<0.001	0.186	<0.001	——	——	0.919	<0.001
Tryglyceride	0.208	<0.001	0.135	<0.001	0.319	<0.001	0.302	<0.001	0.271	<0.001
Cholesterol	0.135	<0.001	0.110	<0.001	0.234	<0.001	0.110	<0.001	0.135	<0.001
Fasting insulin	0.252	<0.001	0.159	<0.001	0.178	<0.001	0.919	<0.001	——	——

BMI:body weight index(weight/height^2^).

In multiple linear regression analysis, liver enzymes as dependent variables, HOMA-IR emerged as a determinant of ALT (β = 0.165, *P*<0.001), AST (β = 0.040, *P*<0.001) and GGT (β = 0.17, *P*<0.001) after adjusting for age, sex, BMI, triglyceride, and cholesterol (Table 3). When further investigating BMI as an independent variable, the associations with liver enzymes attenuated but remained significant (*P*<0.001). The association for HOMA-IR or BMI in relation to liver enzymes became less significant after adjusting for confounding factors.

**Table 3:** Multivariate linear regression analysis for the risk of elevated liver enzymes.

	AST					ALT					GGT				
	β	SE	95% confidence interval		*p value*	β	SE	95% confidence interval		*p value*	β	SE	95% confidence interval		*p value*
			upper bound	lower bound				upper bound	lower bound				upper bound	lower bound	
BMI															
unadjusted	0.011	0.001	0.010	0.012	<0.001	0.033	0.001	0.031	0.035	<0.001	0.040	0.001	0.038	0.042	<0.001
Model 1	0.027	0.001	0.025	0.029	<0.001	0.026	0.001	0.024	0.028	<0.001	0.027	0.001	0.025	0.029	<0.001
Model 2	0.006	0.001	0.005	0.008	<0.001	0.017	0.001	0.015	0.019	<0.001	0.015	0.001	0.012	0.017	<0.001
Model 3	0.006	0.001	0.005	0.007	<0.001	0.015	0.001	0.013	0.017	<0.001	0.011	0.001	0.008	0.013	<0.001
HOMA-IR															
unadjusted	0.105	0.007	0.091	0.118	<0.001	0.352	0.011	0.330	0.373	<0.001	0.432	0.014	0.405	0.459	<0.001
Model 1	0.093	0.007	0.080	0.106	<0.001	0.306	0.010	0.286	0.326	<0.001	0.358	0.012	0.334	0.383	<0.001
Model 2*	0.055	0.008	0.039	0.070	<0.001	0.205	0.012	0.182	0.228	<0.001	0.270	0.014	0.242	0.298	<0.001
Model 3*	0.040	0.008	0.024	0.056	<0.001	0.165	0.012	0.141	0.188	<0.001	0.170	0.014	0.142	0.197	<0.001

Model 1: adjusted for age and sex; Model 2: further adjusted for LogHOMA-IR; Model 3: further adjusted for triglyceride and cholesterol; Model 2*: adjusted for age, sex and body mass index (BMI); Model 3*: further adjusted for triglyceride and cholesterol; β was calculated by multivariate linear regression analysis after adjustment. All variables are log-transformed.

## Discussion

Insulin resistance, as a pathophysiological precursor of type 2 diabetes, is defined as a reduced sensitivity to the action of insulin [19]. Type 2 diabetes is a risk factor for the development of hepatic steatosis and its progression [20]. Numerous studies investigated the relationship between insulin resistance and liver enzymes in individuals diagnosed with type 2 diabetes and NAFLD. Sheng et al. found liver enzymes were associated with HOMA-IR in 212 type 2 diabetes patients with NAFLD [21]. Another study included 95 Chinese patients with newly diagnosed type 2 diabetes found liver enzymes especially ALT, were significantly associated with insulin resistance [22]. Obesity is an important risk factor in developing insulin resistance, type 2 diabetes [23], and a significant predictor of abnormal liver test results [24]. Thus, we evaluated the association of insulin resistance with liver enzymes and the modifying effect of weight status on these associations in non-diabetic Chinese adults. Our study results indicated insulin resistance had significant association with greater risk of elevated ALT, AST and GGT level in non-diabetic adults. Interactions between insulin resistance and weight status were also found played a role in elevating liver enzyme level.

In the multiple linear regression models, HOMA-IR was positively associated with ALT, AST and GGT level after adjustment for age, sex, BMI, cholesterol, and triglyceride. Association between insulin resistance and serum ALT levels has been previously demonstrated. A study conducted in 1732 young adults aged 18–23 years, suggest that insulin resistance is significantly associated with elevated ALT levels, but not with elevated AST levels [25]. Ghamar et al. found the only independent associates of ALT increase in NAFLD patients without diabetes are insulin resistance [26]. Correlations of GGT and ALT with pre-diabetes were determined by characteristics of liver enzymes in a study included 9420 middle-aged Chinese individuals [27]. Our results were consistent with these previous studies. In terms of GGT, Gray et.al presented significant correlations between insulin resistance and GGT in their study examining 40 overweight and obese, non-diabetic adults [23]. Association of insulin resistance with GGT was found in NAFLD subjects after adjusting for effects of major confounding variables [28]. Bradley et al. evaluated continuous associations between GGT and HOMA-IR in 6814 participant, both multivariable linear regression models and linear regression models adjusting risk factors demonstrated positive significant associations between GGT activity and HOMA-IR [29]. Our study confirmed that HOMA-IR is significantly associated with liver enzymes, particularly in ALT and GGT. Although we observed insulin resistance was statistically associated with AST, the coefficient was not as significant as ALT and GGT. It was also reported that AST was not as sensitive or specific to liver damage as ALT and GGT [30, 31].

**Fig 1:**
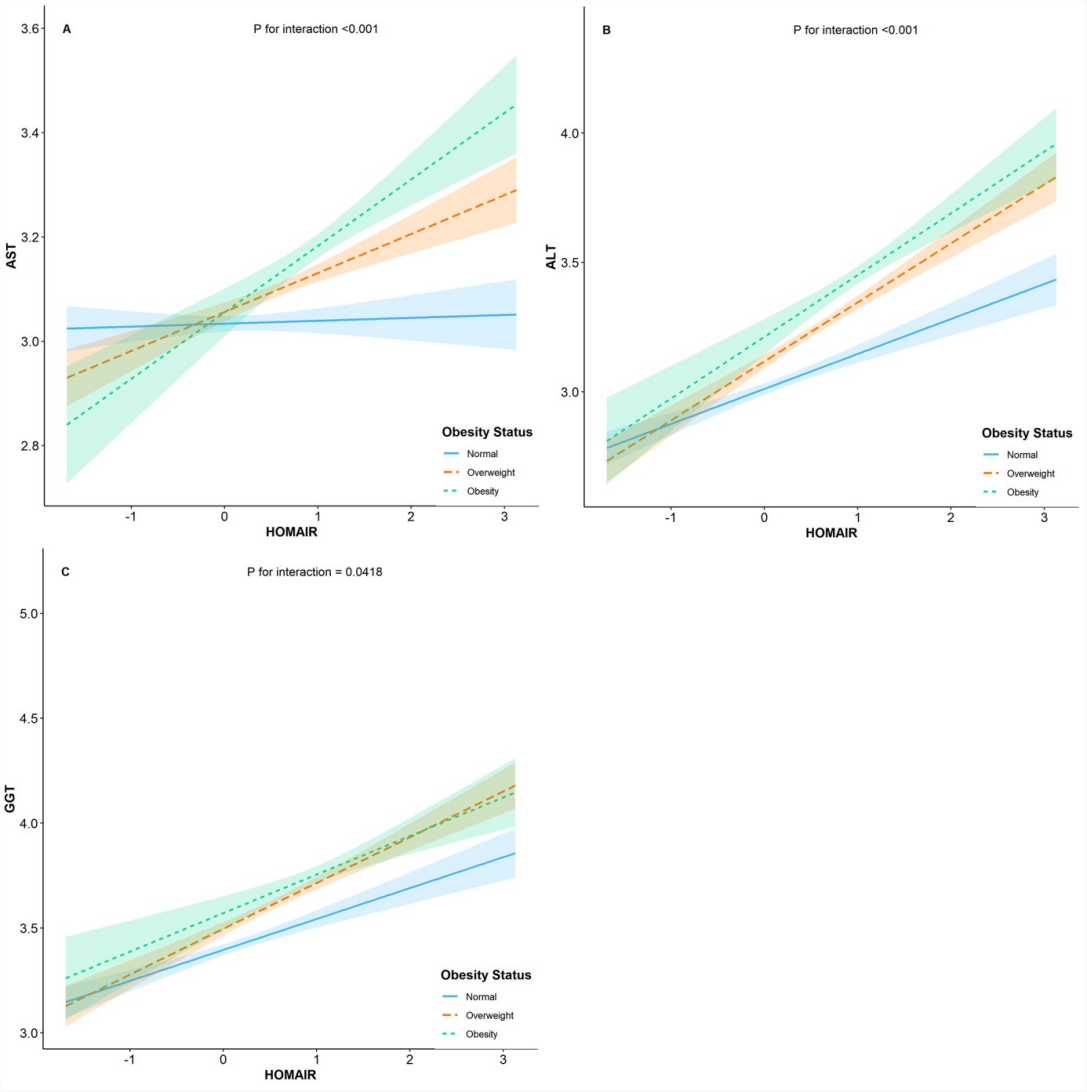
Interaction between weight status and HOMA-IR on liver enzymes.

Since obesity is a well-known contributor to insulin resistance, we further evaluated the relationship of insulin resistance and weight status on the risk of elevated liver enzyme levels. In Fig 1, we observed statistically significant interactions between weight status and insulin resistance in association with elevated liver enzymes, which was not consistent with previous studies. Wallance et al. found GGT but not ALT or AST levels are inversely associated with insulin sensitivity independently of central obesity in non-diabetic men, while in women GGT levels were positively related to intra-abdominal fat area and waist-to-hip ratio but were not related to insulin sensitivity [6]. Other cross-sectional study investigating Hispanic/Latino youths observed statistically significant interactions between BMI and insulin resistance in association with elevated liver enzymes, and this association was partially mediated by inflammation and endothelial dysfunction [31]. The inconsistency of study findings was possibly due to sample sizes, ethnics, and adjustment for confounding factors.

The mechanisms underlying the association between insulin resistance and liver dysfunction may be explained by the double hit hypothesis. The first hit produces steatosis; the second hit requires sources of oxidative stress, such as induction of CYP2E1 by a high-fat diet and an increase in the intrahepatic concentration of free fatty acids, which initiate significant lipid peroxidation [32]. Individuals with obesity and NAFLD exhibit high levels of de novo lipogenesis (DNL) in both fasting and postprandial state [33, 34] and the anti-lipolytic effects of insulin are diminished by adipose tissue; that is, the rate of basal lypolysis in adipocytes is increased, which increase the delivery of fatty acids to the liver [35, 36]. In addition to increase hepatic lipid deposition, insulin resistance exacerbate liver oxidative stress by upregulating CYP2E1 [32]. Obesity, as the major contributor of insulin resistance, amplifies the detrimental effects of insulin resistance on the liver function. Meanwhile, obesity seems to play a role in the progression from steatosis to steatohepatitis due to dysregulation of adipokines, dysbiosis of the gut microbiota and chronic inflammation [37], which possibly explain the modifying effect of weight status on the associations between insulin resistance and liver enzymes in our study.

To our knowledge, this is the first study to evaluate the interaction between insulin resistance and weight status on liver function in a large Chinese population. Annual health check-up is trending in general population in relation to early disease diagnosis and health promotion in China. Identifying overweight/obese individuals with insulin resistance could help detect those who are at highest risk for developing steatosis. As China has numerous overweight/obese individuals as well as NAFLD, our results may have positive effects on the early diagnosis of hepatic disease and thus promote early treatment.

The present study has some limitations. First, since this is a cross-sectional study, the causal relationship of insulin resistance with weight status on the liver enzymes could not be inferred. Second, the study subjects were from an annual health check-up center and might therefore be more health conscious and wealthier, and it remains unknown whether our results could be extrapolated to other ethnics.

In summary, out study demonstrates that in non-diabetic individuals, insulin resistance is a significant predictive factor for abnormal liver enzymes, and these associations were stronger in overweight/obese population. If this finding is confirmed in future studies, evaluation of insulin sensitivity and weight status may help detect those who are at highest risk for developing abnormal liver function, and management of insulin resistance through lifestyle intervention or medication, especially in overweight/obese population, may be a promising way to prevent disease progression.
